# Is rifaximin better than nonabsorbable disaccharides in hepatic encephalopathy? A meta-analysis

**DOI:** 10.1097/MD.0000000000028232

**Published:** 2021-12-23

**Authors:** Junxiong Cheng, Yafang Chen, Wenfu Cao, Guoqing Zuo

**Affiliations:** a College of Traditional Chinese Medicine, Chongqing Medical University, Chongqing Key Laboratory of Traditional Chinese Medicine for Prevention and Cure of Metabolic Diseases, Chongqing, PR China; b College of Pharmaceutical Sclences and Chinese Medicine, Southwest University, Chongqing, PR China; c Department of Gastroenterology, Chongqing Hospital of Traditional Chinese Medicine, Chongqing, PR China.

**Keywords:** hepatic encephalopathy, lactitol, lactulose, nonabsorbable disaccharides, rifaximin

## Abstract

**Background:**

The purpose of the present meta-analysis was to compare the efficacy of rifaximin and nonabsorbable disaccharides (NADs) in hepatic encephalopathy (HE).

**Methods:**

After the registration of the present meta-analysis on INPLASY, all procedures were performed according to PRISMA 2020. Relevant literature was retrieved on PubMed, Embase, and the Cochrane Library up to September 5, 2021. The Newcastle-Ottawa Scale (NOS) was used to assess the quality of the enrolled studies, and Review Manager software (version 5.3) was used to analyze the clinical efficacy, blood ammonia and adverse effects.

**Results:**

Six studies with 559 patients were included in the present meta-analysis. There were no significant differences in the basic characteristics of the included studies. Analysis of the complete resolution of HE showed that rifaximin was better than NADs (risk ratio [RR] = 1.87, 95% confidence interval [CI] = 1.03–3.39, *P* = .04). However, there were no significant differences in mental status (RR = 1.04, 95% CI = 0.92–1.18, *P* = .53), blood ammonia level (standard mean difference = −0.02, 95% CI = −0.40–0.02, *P* = .08), or drug adverse drug effects (OR = 0.43, 95% CI = 0.10–1.77, *I*^2^ = 56%, *P* = .24) between the rifaximin and NADs treatment groups.

**Conclusion:**

Rifaximin is not superior to NADs in the treatment of HE.

## 1 Introduction

Hepatic encephalopathy (HE) is a neuropsychiatric disease caused by liver dysfunction/failure, or portosystemic bypass and is characterized by alterations in cognition, personality, and consciousness. Thus, HE has been classified as type A, B, or, C by etiology and divided into grades I to IV according to the mental status of the patients.^[1]^ Patients with liver cirrhosis and neuropsychological or neurophysiological alterations (without clinical symptoms of HE) were considered to have minimal hepatic encephalopathy (MHE).^[2]^ Recently, HE was also categorized into convert HE(MHE and grade I) and overt HE (II, III, IV grade).^[3]^

Although the exact mechanism of HE is still under debate, scholars have proposed several hypotheses, including an ammonia poisoning theory. After passing through the blood-brain barrier, ammonia mainly enters astrocytes, but due to its physical and chemical properties, ammonia damages cells and affects intracellular biochemical reactions.^[4]^ Therefore, the level of ammonia in patients is also a major factor affecting the severity of HE.

Rifaximin, a derivative of the antibiotic rifamycin, inhibits the RNA synthesis when combined with the β subunit of the DNA-dependent RNA polymerase in bacteria^[5]^ and previous studies have found that rifaximin can improve the clinical symptoms of HE with few side effects.^[6]^ This has attracted more attention than other anti-HE drugs. Nonabsorbable disaccharides (NADs), mainly lactulose and lactitol, have been used earlier in the treatment of HE.^[7]^ Lactulose is a synthetic derivative of lactose, that is broken down in the colon by saccharolytic bacteria. This leads to an acidic environment due to the production of lactic acid, and is probably the mechanism of the action of lactulose in the treatment of HE.

Previous studies have suggested that both rifaximin and NADs are effective for HE, and the former has fewer adverse drug effects than NADs.^[8]^ They are recommended for HE in guidelines, with lactulose as the first line and rifaximin as an alternative.^[9,10]^ However, some studies have indicated that rifaximin is better than NADs.^[8,11]^ These contradictory views are not conducive for HE treatment. Therefore, we conducted a meta-analysis to determine whether rifaximin is better than NADs for the treatment of HE.

## 2 Methods

This study was performed according to the Preferred Reporting Items for Systematic reviews and Meta-Analyses (PRISMA) statement and registered with INPLASY (registration number: INPLASY202180094). This study is a non-clinical study, so ethical approval is not applicable.

### 2.1 Search strategies

A systematic and comprehensive literature search was conducted on the PubMed, Cochrane Library and Embase databases up to September 5, 2021. The key words used for search strategies were as follows: cirrhosis, hepatic encephalopathy, rifaximin, NADs, lactulose, or lactitol and their relative Medical Subject Heading (MeSH) terms. The strategy we used for PubMed is as follows: (((“Rifaximin” [Mesh]) OR (((((((((((Rifaximin [Title/Abstract]) OR (4-Deoxy-4’-methylpyrido(1’,2’-1,2)imidazo(5,4C)rifamycin [Title/Abstract])) OR (L 105 [Title/Abstract])) OR (L-105 [Title/Abstract])) OR (L105 [Title/Abstract])) OR (Redactiv [Title/Abstract])) OR (Xifaxan [Title/Abstract])) OR (rifaximin α [Title/Abstract])) OR (rifaximin-α [Title/Abstract])) OR (rifaximin aerfa [Title/Abstract])) OR (rifaximin-aerfa [Title/Abstract]))) AND ((((((“Lactulose” [Mesh]) OR ((((Lactulose [Title/Abstract]) OR (Duphalac [Title/Abstract])) OR (Normase [Title/Abstract])) OR (Amivalex [Title/Abstract]))) OR (“lactitol” [Supplementary Concept])) OR ((((((lactitol [Title/Abstract]) OR (4-O-beta-D-galactopyranosyl-D-glucitol [Title/Abstract])) OR (Oponaf [Title/Abstract])) OR (Emportal [Title/Abstract])) OR (Neda Lactiv Importal [Title/Abstract])) OR (Importal [Title/Abstract]))) OR (“Disaccharidess” [Mesh])) OR ((Disaccharidess [Title/Abstract]) OR (Disaccharides [Title/Abstract])))) AND ((“Hepatic Encephalopathy” [Mesh]) OR (((((((((((((((((((((((((((Hepatic Encephalopathy [Title/Abstract]) OR (Encephalopathies, Hepatic [Title/Abstract])) OR (Hepatic Encephalopathies [Title/Abstract])) OR (Encephalopathy, Hepatic [Title/Abstract])) OR (Portal-Systemic Encephalopathy [Title/Abstract])) OR (Portal Systemic Encephalopathy [Title/Abstract])) OR (Encephalopathy, Portal-Systemic [Title/Abstract])) OR (Encephalopathies, Portal-Systemic [Title/Abstract])) OR (Encephalopathy, Portal Systemic [Title/Abstract])) OR (Portal-Systemic Encephalopathies [Title/Abstract])) OR (Encephalopathy, Portosystemic [Title/Abstract])) OR (Hepatocerebral Encephalopathy [Title/Abstract])) OR (Portosystemic Encephalopathy [Title/Abstract])) OR (Encephalopathies, Portosystemic [Title/Abstract])) OR (Portosystemic Encephalopathies [Title/Abstract])) OR (Encephalopathy, Hepatocerebral [Title/Abstract])) OR (Encephalopathies, Hepatocerebral [Title/Abstract])) OR (Hepatocerebral Encephalopathies [Title/Abstract])) OR (Hepatic Coma [Title/Abstract])) OR (Coma, Hepatic [Title/Abstract])) OR (Comas, Hepatic [Title/Abstract])) OR (Hepatic Comas [Title/Abstract])) OR (Hepatic Stupor [Title/Abstract])) OR (Hepatic Stupors [Title/Abstract])) OR (Stupor, Hepatic [Title/Abstract])) OR (Stupors, Hepatic [Title/Abstract])) OR (Fulminant Hepatic Failure with Cerebral Edema [Title/Abstract]))).

### 2.2 Selection criteria

Two authors independently reviewed the titles and abstracts after the search process. The 2 reviewers had a discussion if a disagreement occurred, and a third reviewer joined to decide according to the full text if the discussion did not reach a goal. Duplicated articles, case reports, editorials, commentaries, letters, review articles, animal studies and guidelines were excluded from the present study.

Studies that met all the inclusion criteria were included:

1. randomized clinical trials;
2. studies in which patients were divided into at least 2 groups, each treated with rifaximin and NADs respectively;
3. and studies that reported the endpoints related to the present meta-analysis, including mental status, complete resolution or HE grade reduced to zero, ammonia level, and therapy-related adverse effects.

The exclusion criteria were as follows:

1. studies in which the HE developed in patients was not secondary to cirrhosis;
2. studies in which rifaximin and/or NADs treatment was combined with other drugs; and
3. studies in which no endpoint was met in the present meta-analysis.

### 2.3 Data extraction

Data were extracted by 2 reviewers independently, and the extracted data were as follows: the first author's name, year of publication, country, study design, duration, total number of participants, Child-Turcotte-Pugh (CTP) class, the type of HE, loss of follow-up, interventions and relevant outcomes.

### 2.4 Statistical analysis

The bias risk of each study was assessed using the Review Manager 5.3. The risk ratio (RR) with 95% confidence interval (CI) was adopted to analyze dichotomous variables, and the standard mean difference with 95% CI was used for continuous variables, and the heterogeneity of the data included in the study was evaluated using the *I*-square. When the *I*-square was >50%, it indicated that the results of each study were highly heterogeneous, and the random effect model was used for analysis; otherwise, the fixed effect model (Mantel–Haenszel method) was adopted. The blood ammonia values were converted to standard mean deviation before the analysis, if necessary, the number of effective patients was converted from the relevant percentage in each article. A forest map was used to show the results of the meta-analysis, and a funnel map was used to observe publication bias in the results of the meta-analysis. Differences were considered statistically significant at *P* < .05.

## 3 Results

### 3.1 Characteristics of the included studies

**Figure 1:**
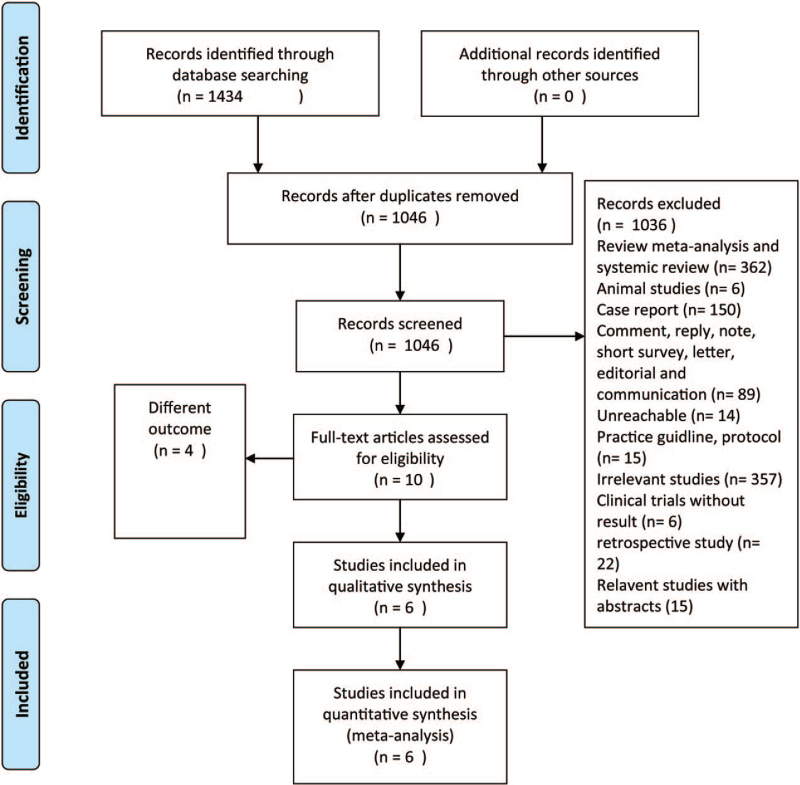
The flow chart of present meta-analysis. All articles were singly excluded.

We obtained 1434 articles by retrieving the three databases, including 167 from the Chocrane Library, 1013 from the EMBASE database and 254 from PubMed. After screening the title and abstract, repetitive and irrelevant articles were excluded, 10 articles were reviewed in full text, and finally 6 articles were included in the present meta-analysis. A flow chart of the article screening process is shown in Figure 1.

The basic characteristics of patients and clinical data presented in the 6 articles are summarized in Tables 1 and 2. A total of 284 patients treated with rifaximin and 275 patients treated with NADs were included in this meta-analysis.

**Table 1:** Characteristics of the studies included in present meta-analysis.

Authors, year	Country	Duration	Included patients (Rifaximin Vs. NADs) (n, male: female)	Type of HE	Child-Pugh class (A/B/C), rifaximin vs NADs	Lost to follow up(Rifaximin /NADs)	Interventions	Outcomes
Mas, 2003^[12]^	Spain	5/10 d	33: 17 vs 39: 14	HE (I-III grade)	N	8/7	Rifaximin 200 mg, 3 times/d vs Lactitol 20 g, 3 times/d	HE improvement, ammonia level, adverse effects,
Paik, 2005^[13]^	Korea	7 d	24: 8 vs 13: 9	HE (I-III grade)	0/16/16 vs 0/14/8	0/0	Rifaximin 1200 mg/d vs lactulose 90 mL/d	HE improvement, ammonia level, adverse effects,
Wahib, 2014^[14]^	Egypt	7 d	25 vs 25	HE (I-III grade)	N	0/0	Rifaximin 400 mg, 3 times/d vs lactulose 30 mL, 3 times/day	HE resolution, ammonia level
Sidhu, 2016^[15]^	India	3 mo	45: 12 vs 39: 16	MHE	N	0/0	Rifaximin 400 mg, 3 times/d vs Lactulose 30–120 mL/d	HE improvement
Suzuki, 2018^[8]^	Japan	14 d	43: 41 vs 46: 41	HE (I-II grade)	12/55/17 vs 10/57/20	6/5	Rifaximin 400 mg, 3 times/day vs. lactitol 6–12 g, 3 times/d	Ammonia level, adverse effects,
Pawar, 2019^[11]^	India	3 mo	43: 3 vs 31: 4	MHE	11/20/6 vs 12/17/6	0/0	Rifaximin 550 mg, 2 times/d vs lactulose 30–60 g /d	HE improvement, adverse effects,

HE = hepatic encephalopathy, MHE = minimal hepatic encephalopathy, N = not metioned, NADs = nonabsorbable disaccharides, including lactulose and lactitol.

**Table 2:** Clinical data of the studies included in present meta-analysis.

			Blood ammonia level (pretreatment: final)		
Authors, year	HE improvement (Rifaximin vs NADs,alteration: total)	HE resolution (Rifaximin/NADs, alteration: total)	Rifaximin	Rifaximin	Adverse drug reactions (Rifaximin/NADs, alteration: total)
Mas, 2003^[12]^	40:49 vs 41:51	26:49/19:51	120.5 (12.1–300): 69.5 (13–268) g/dL	120.5 (12.1–300): 69.5 (13–268) g/dl	3: 49 vs 2: 51
Paik, 2005^[13]^	26:32 vs 16:22	N	192.7 ± 63.4: 128.3 ± 49.1 mmol/L	192.7 ± 63.4: 128.3 ± 49.1 mmol/L	1: 32 vs 1:22
Wahib, 2014^[14]^	N	21:25/8:25	N	N	N
Sidhu, 2016^[15]^	42:57 vs 38:55	N	134.9 (49.2): 119.5 (59.5) μg/dL	134.9 (49.2): 119.5 (59.5) μg/dL	N
Suzuki, 2018^[8]^	N	N	179.400 ± 19.570: 135.760 ± 21.423 μmol/l	179.400 ± 19.570: 135.760 ± 21.423 μmol/l	5: 84 vs 12: 87
Pawar, 2019^[11]^	26:37 vs 25:35	N	N	N	0:37 vs 15:35

HE = hepatic encephalopathy, NADs = nonabsorbable disaccharides, including lactulose and lactitol, N = not mentioned.

### 3.2 Evaluation of risk bias

**Figure 2:**
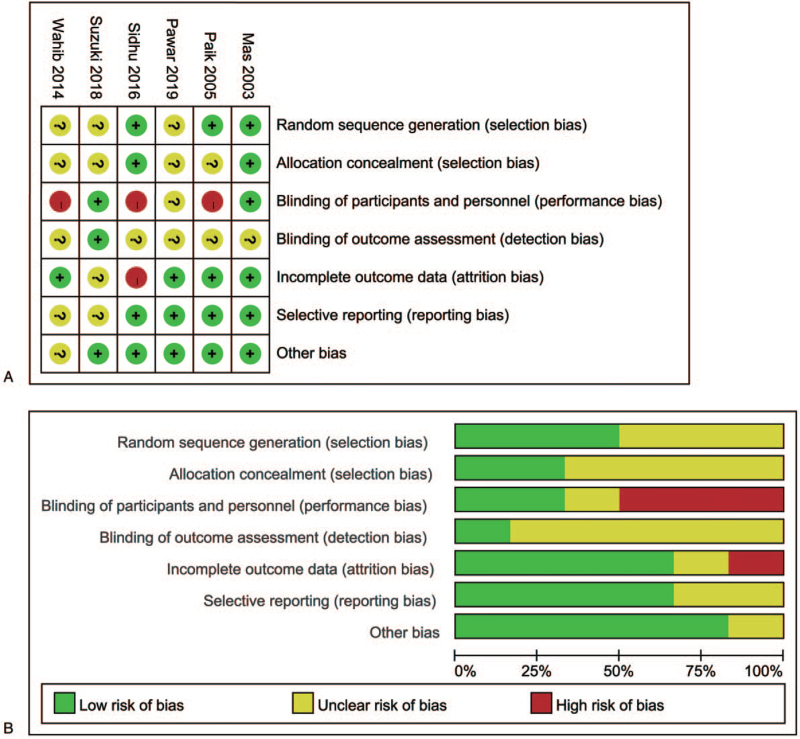
Risk of bias summary (A) and risk of bias graph (B). Reviewers’ judgements about each risk of bias item presented as percentages across all included studies.

The included studies were from diverse countries, including Spain, Korea, Egypt, India and Japan. The publication year ranged from 2003 to 2019. Three articles described the randomized sequence generation, and only 2 articles described allocation concealment. As many studies have provided informed consent, we rated it as a high risk of performance bias. Most studies showed a low risk of attrition, reporting or other biases. The risk of bias summary and risk of bias graph of the 6 studies are summarized in Figure 2.

### 3.3 Mental status

**Figure 3:**
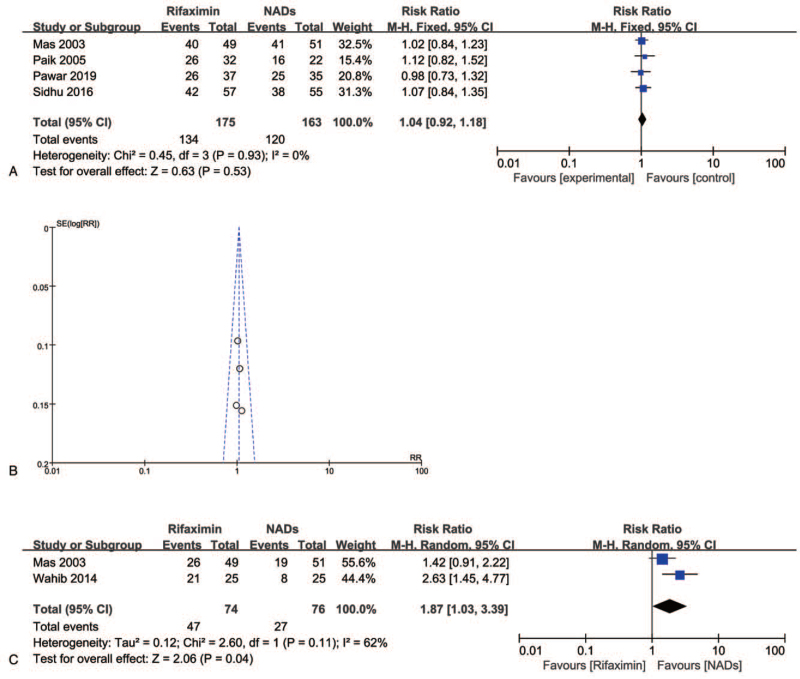
Forest plot of mental status improvement of rifaximin and NADs treated patients. Forest map (A) and funnel map (B) of the 4 studies, and complete resolution of mental status in 2 studies (C).

Four studies reported the mental status of patients including both HE and MHE. In total, 175 patients were treated with rifaximin, and 163 patients were treated with NADs. 134 and 120 patients achieved relief from HE in the 2 groups, respectively. The forest plot showed that there was very low heterogeneity among them (*I*^2^ = 0%); however, there was no significant difference between the rifaximin-treated group and the NADs-treated group (RR = 1.04, 95% CI = 0.92–1.18, *P* = .53) (Fig. 3A). The funnel map indicate a low heterogeneity among the 4 studies (Fig. 3B).

We are also concerned about another question: is there any difference between the 2 groups in terms of complete resolution of HE or a reduction in HE grade after treatment? However, only 2 studies mentioned this. The random effect analysis of the risk ratio was performed as the heterogeneity was 62%, and results indicate that rifaximin is a more effective drug than NADs (RR = 1.87, 95% CI = 1.03–3.39, *P* = .04) (Fig. 3C).

### 3.4 Blood ammonia

**Figure 4:**
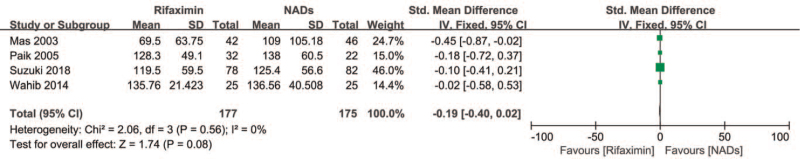
Forest plot of blood ammonia variation.

As shown in Figure 4, 4 studies mentioned the blood ammonia level. As different units were used in each study, the standard mean deviation was used to analyze the differences after the extraction of blood ammonia data. Data analysis indicate a low heterogeneity (*I*^2^ = 0%) and rifaximin was not better than NADs in terms of reducing blood ammonia levels (standard mean difference = −0.02, 95% CI = −0.40–0.02, *P* = .08) (Fig. 4).

### 3.5 Adverse effect

**Figure 5:**
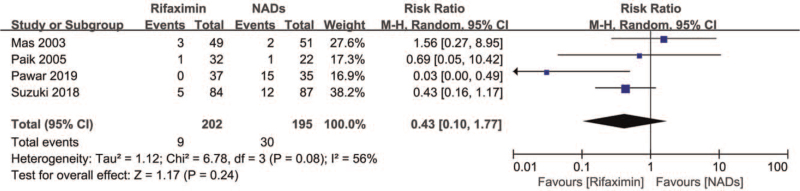
Forest plot of adverse drug effect.

Four studies investigated adverse drug effects, and there was no significant difference between the 2 groups (RR = 0.43, 95% CI = 0.10–1.77, *I*^2^ = 56%, *P* = .24) (Fig. 5**)**. However, due to the lack of statistics on different adverse effects, such as diarrhea and abdominal pain, we did not analyze the differences in the occurrence of specific adverse effects.

## 4 Discussion

The present meta-analysis was conducted to compare the efficacy of rifaximin and NADs in the treatment of HE. Two previous meta-analyses were not strict enough in the review of included articles.^[12,13]^ Articles in which rifaximin was in combination with other drugs, andconference publications with only abstracts were also included in their studies, which could not accurately evaluate the efficacy of rifaximin. The present study strictly reviewed the included studies, which ensured the credibility of this study, and the effects of rifaximin and NADs in HE were analyzed using appropriate statistical methods. Our results indicate that rifaximin has no obvious advantage over NADs in the treatment of HE, although clinical studies in recent years have shown the advantage of rifaximin. Simultaneously, blood ammonia levels and adverse drug effects did not differ between the 2 drugs. Interestingly, rifaximin was better than NADs in the complete resolution of HE. The contradiction may be due to the following: first, inconsistent duration of the included studies, which ranged from 7 days to 3 months. A previous study showed that efficiency would increase significantly with a longer duration,^[14]^ which may lead to differences in the same treatment group between each study. Second, lactulose or lactitol aims to ensure patients 2 to 3 semi-formed stools per day, so the dose is not constricted, and could be 60 to 120 g/day, however, it was recommended 5 to 30 mL each time, and 2 to 3 times/day according to the guidelines for HE.^[15]^ Finally, the complete resolution results were calculated from 2 studies that only enrolled patients with overt HE (grade ≥1) and without MHE.^[16,17]^ The methods for diagnosis of MHE are still under development, and none of them can cover the complexity of cognitive impairment in MHE.^[18]^ Thus, there may be some differences in the confirmation of therapeutic effectiveness.

In addition, the cost of treatment should be considered. To our knowledge, rifaximin is more expensive than lactulose or lactitol. It seems that NADs are more suitable for clinical use as rifaximin does not show significantly better effect than NADs. To date, there is still a lack of cost-effectiveness studies comparing rifaximin and NADs in the treatment of HE. Only the combined use of rifaximin was evaluated, which indicated that rifaximin 550 mg twice daily is a cost savings plan for the treatment of HE.^[19,20]^ Recent studies have pointed out that the total cost of hospitalization in patients with HE treated with rifaximin is not higher than that in the lactulose treatment group, indicating the high application potential of rifaximin in HE.^[21]^

The long-term use of rifaximin can potentially lead to resistance, which may limit its use. Previous studies revealed that 2 types of bacteria, Staphylococci and Clostridium difficile (C. difficile), showed resistance to antibiotics after long-term usage of rifaximin. It was also found that patients with liver cirrhosis tend to develop drug-resistant Staphylococci after the administration of rifamycins. However, the infection tends to disappear in about half of them.^[22]^ Another study suggests that the use of rifaximin be avoided in patients at risk of staphylococcal infections.^[23]^ Although infections with rifamycin-resistant strains of C. difficile tend to increase in patients with the use of rifamycins, including rifaximin and rifampin, the correlation remains unclear.^[24]^ A recent study showed that prior therapy with rifamycins is a significant risk factor for developing rifaximin-resistant C. difficile strain infections.^[25]^ However, a recent randomized clinical trial indicated that rifaximin does not lead to antimicrobial resistance.^[26]^ These results, therefore, give clinicians the freedom of choice to administer rifaximin in HE until further research is conducted.

We acknowledge that there are limitations to our study. Some articles included in this paper did not describe the methods of the clinical trials in adequate detail. Second, the interventions were not completely consistent in the included studies, such as the drug dose and duration of treatment. Moreover, many old studies were unavailable (or withdrawn), and the total number of studies and patients included in this study was very small.

In conclusion, we cautiously propose that rifaximin should be used as a first-line treatment for overt HE and that its usage as a second-line treatment for MHE be continued, if rifaximin-related microbial resistance is of low risk. Additionally, lactulose should still be used as the first-line treatment of MHE. Further randomized clinical trials, especially those with high-quality, large samples and in which antimicrobial resistance is studied, are required to verify our suggestions.

## Acknowledgments

We thank Li Shichao for his suggestions.

## Author contributions

**Conceptualization:** Junxiong Cheng, Wenfu Cao.

**Data curation:** Junxiong Cheng, Yafang Chen.

**Software:** Junxiong Cheng, Yafang Chen.

**Supervision:** Yafang Chen, Guoqing Zuo.

**Validation:** Junxiong Cheng.

**Writing – original draft:** Junxiong Cheng.

**Writing – review & editing:** Guoqing Zuo.
